# Towards femtosecond on-chip electronics based on plasmonic hot electron nano-emitters

**DOI:** 10.1038/s41467-018-04666-y

**Published:** 2018-06-25

**Authors:** Christoph Karnetzky, Philipp Zimmermann, Christopher Trummer, Carolina Duque Sierra, Martin Wörle, Reinhard Kienberger, Alexander Holleitner

**Affiliations:** 1 Walter Schottky Institute and Physics Department, Technical University of Munich, Am Coulombwall 4a, 85748 Garching, Germany; 2 Nanosystems Initiative Munich (NIM), Schellingstr. 4, 80799 Munich, Germany; 3 Physik-Department E11, Technical University of Munich, James-Franck-Str. 1, 85748 Garching, Germany; 4 Max-Planck-Institut für Quantenoptik, Hans Kopfermann-Straße 1, 85748 Garching, Germany

## Abstract

To combine the advantages of ultrafast femtosecond nano-optics with an on-chip communication scheme, optical signals with a frequency of several hundreds of THz need to be down-converted to coherent electronic signals propagating on-chip. So far, this has not been achieved because of the overall slow response time of nanoscale electronic circuits. Here, we demonstrate that 14 fs optical pulses in the near-infrared can drive electronic on-chip circuits with a prospective bandwidth up to 10 THz. The corresponding electronic pulses propagate in macroscopic striplines on a millimeter scale. We exploit femtosecond photoswitches based on asymmetric, nanoscale metal junctions to drive the pulses. The non-linear ultrafast response is based on a plasmonically enhanced, multiphoton absorption resulting in a field emission of ballistic hot electrons propagating across the nanoscale junctions. Our results pave the way towards femtosecond electronics integrated in wafer-scale THz circuits.

## Abstract (web summary)

For future ultrafast opto-electronic circuits, optical signals must be interfaced with coherent electronic signals. The authors develop asymmetric plasmonic nanojunctions that convert fs light pulses to THz electronic transients that can propagate on-chip for up to a mm, enabling such an interface.

## Introduction

Recent work on the photoemission at sharp metal tips in vacuum suggests that the electron emission process can be separated into different regimes, starting with a multiphoton emission for moderate optical fields^1–4^, and two limiting cases of the so-called strong-field photoelectron emission^5–7^. The latter two are characterized by the electron quiver amplitude which is either smaller (quiver regime) or larger (sub-cycle photoemission regime) than the characteristic decay length of the optical near-field at the metal surfaces^5–7^. Photoemission processes at moderate optical fields have also been shown to be capable of driving ultrafast currents in nanoscale circuits defined on substrates^8–10^. In all cases, the electron emission can be significantly increased by plasmonic effects, for instance, in resonant plasmonic nanostructures such as bowtie antennas^9–14^. However, driving a dc unipolar current in such nanostructures requires a symmetry breaking of the spatio-temporal electron dynamics^15,16^. For nanostructures, this has been achieved by using few-cycle femtosecond pulses for the optical excitation^8,9^ or by applying strong dc electric fields at the emitter electrodes^10^. The electrodynamic symmetry breaking also favors optical rectification processes, which are typically detectable at the lowest optical intensities for metallic nanostructures^13,14^.

Here, we show that by using asymmetric plasmonic nanojunctions instead of symmetric ones, we are able to achieve the required symmetry breaking to induce a unipolar photoemission of hot electrons without applying a voltage at moderate optical intensities of near-infrared (NIR) femtosecond pulses. Our findings suggest that the detected non-linear photoemission currents comprise electrons traveling ballistically across the nanojunctions in vacuum. At the lowest optical intensities, our results are consistent with an optical rectification process at the metallic surfaces. Most importantly, we verify that the demonstrated technology can be utilized for ultrafast photoswitches driving on-chip THz circuits. We prove a coherent propagation of corresponding on-chip THz transients by an Auston switch technology^17^. The ultimate switching time in the asymmetric nanojunctions is limited by the laser pulse duration and the time of flight of the ballistic electrons, which was reported to be as fast as 900 as for a 8 nm junction gap^9^. This (sub-) femtosecond timescale outperforms known Auston photoswitch technologies based on non-radiative carrier capture sites in semiconductors by two orders of magnitude^17–23^. Our work reveals that the photoemission dynamics in asymmetric nanojunctions allow to convert a femtosecond NIR-optical pulse into a coherent on-chip signal in the THz range. In this respect, we expand on-chip electronics from GHz up to several THz into the so-called THz Gap between electronic and optical applications^24^. The THz pulses are driven by the non-linear photoemission response of the asymmetric nanojunctions, and are coupled into macroscopic co-planar striplines by near-field interactions. The on-chip signal propagation along the striplines extends up to several hundreds of micrometers. Insofar, we show that femtosecond electronics based on asymmetric nanoscale junctions may prove useful for on-chip clock and synchronization dynamics up to 10 THz and to realize a macroscopic on-chip signal transduction on a femtosecond timescale. For the few-femtosecond control pulses, we utilize a compact Er:fiber technology^25^, such that the demonstrated coherent electronics is fully telecom compatible.

## Results

### Asymmetric nanojunctions centered in THz stripline circuits

**Fig. 1:**
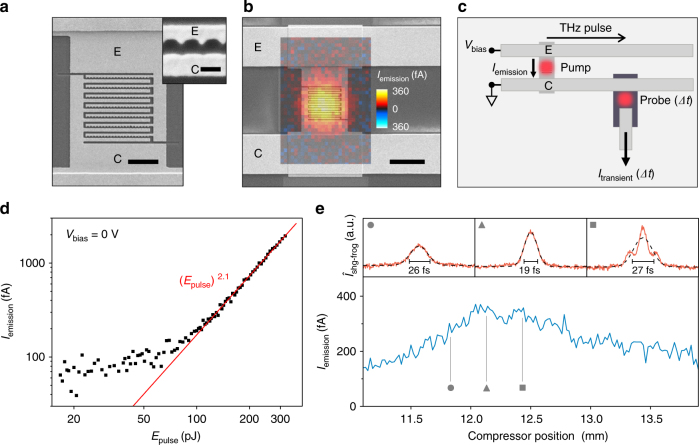
Femtosecond photoemission in nanoscale junctions and THz on-chip circuits. **a** Scanning electron microscope (SEM) image of Ti/Au contacts and asymmetric nanojunctions with the emitter (collector) denoted as ‘E’ (‘C’). Scale bar, 2 µm. Inset: Close-up showing the asymmetry between the emitter and collector. Scale bar, 200 nm. **b** Lateral map of the unipolar photoemission current *I*_emission_ at zero bias *V*_bias_ across asymmetric nanojunctions (overlaid plot), which are contacted by two Ti/Au striplines (outer SEM image). Scale bar, 5 µm. **c** Schematic of the on-chip THz time-domain circuit with optical femtosecond pump and probe pulses triggering the electronic read-out. *I*_emission_ describes the time-integrated current, while *I*_transient_ captures the time-resolved electromagnetic transients in the striplines at a time delay *Δt*. In all shown experiments, *V*_bias_ = 0 V. **d** Non-linear *I*_emission_ vs laser pulse energy *E*_pulse_ with a power law fit (red line). **e** Lower graph shows *I*_emission_ vs laser compressor position at a fixed *E*_pulse_ = 150 pJ. The three upper insets show the second-harmonic generation frequency-resolved optical gating (SHG-FROG) intensity *Î*_shg-frog_ for three given compressor positions denoted by a circle, triangle, and square. All measurements are performed at 77 K and in vacuum

We fabricate asymmetric nanojunctions by focused-ion beam (FIB) milling of a 35 nm thick Au layer on a sapphire substrate with 2 nm Ti as an adhesion layer. Each nanojunction consists of a triangular-shaped emitter (‘E’) electrode and a plane collector (‘C’) electrode separated by a vacuum gap with a distance of *d*_gap_ ~ 90 nm (Fig. 1a). The nanojunctions are positioned in between two co-planar THz striplines made from Ti/Au with the emitter and the collector electrodes directly connected to the striplines (Fig. 1b). We use NIR broadband pulses with a photon energy *E*_pump_ = (0.9–1.3) eV as an excitation of a photocurrent (cf. Supplementary Fig. 1). The time-integrated photocurrent *I*_emission_ is measured between the striplines at zero bias (*V*_bias_ = 0 V) (Fig. 1c). For time-resolved measurements, an additional NIR probe pulse at *E*_probe_ = 1.59 eV with a pulse duration of 100 fs full width at half maximum (FWHM) triggers a semiconductor photoswitch for the electronic read-out with a switching time of about 500 fs^21–23^. All measurements are performed at 77 K in vacuum.

Figure 1b depicts a scanning electron microscope (SEM) image of the striplines with the nanojunctions located in the center. The graph is overlaid with a map of *I*_emission_ of the same area. We find that the maximum current is located at the position of the nanojunctions (cf. Supplementary Fig. 2). The photocurrent *I*_emission_ is unipolar, and its polarity is such that electrons propagate from the emitter to the collector with an amplitude of 360 fA. We note that the spatial extension of *I*_emission_ is significantly smaller than the laser spot of ~7.5 µm (FWHM) (cf. Supplementary Fig. 2) pointing towards a super-linear intensity dependence. Consistently, Fig. 1d shows that *I*_emission_ follows a power law (*E*_pulse_)^*β*^ with a fitted power coefficient in the range of 2 ≤ *β* ≤ 3 for the investigated samples. Such coefficients are typically explained by multiphoton processes as discussed below^1–4,6,7,9,10,13,14,16^.

We use an SF10-prism compressor to control the temporo-spatial shape of the pump pulse and a second-harmonic generation frequency-resolved optical gating technique (SHG-FROG) for its characterization^26^. The upper panels of Fig. 1e show the second-harmonic-intensity *Î*_shg-frog_ vs time delay for three different compressor settings. Gaussian fits yield a FWHM between 19 fs (triangle) and 26 fs (circle), up to ~27 fs for non-Gaussian pulses (square). The width of the shortest pulse (triangle) translates to a temporal FWHM of 14 fs (cf. Supplementary Fig. 1). The lower panel of Fig. 1e shows the corresponding emission current *I*_emission_ across the asymmetric nanojunctions for the investigated range of compressor positions. We observe a maximum *I*_emission_ for the shortest laser pulses, while the pulse energy is constant for all compressor positions (*E*_pulse_ = 150 pJ). Figure 1e demonstrates that *I*_emission_ depends predominantly on the electric field of the impinging photons instead of the average laser intensity.

### Photoemission processes in the nanojunctions

**Fig. 2:**
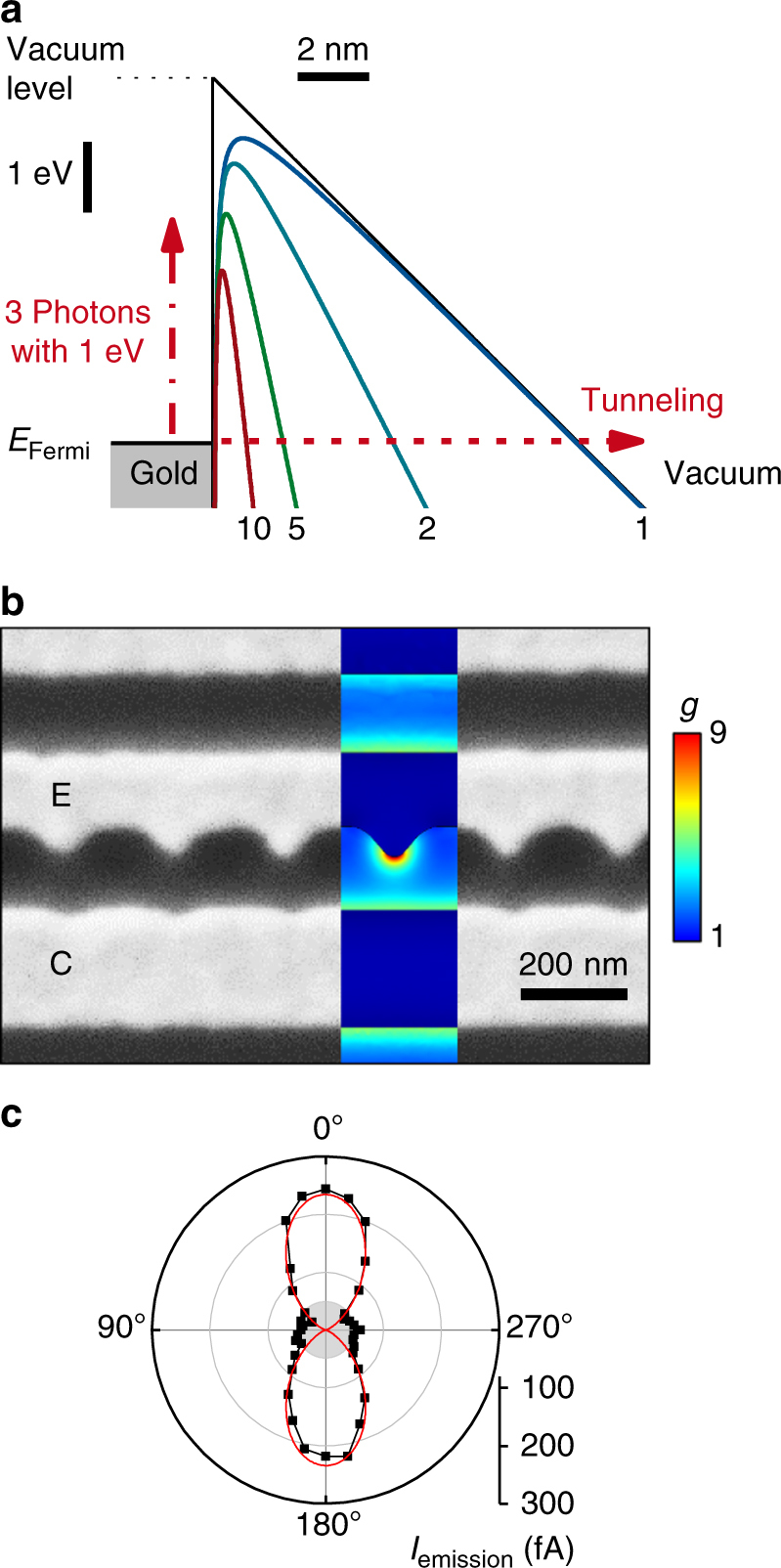
Asymmetric nanoscale junctions for plasmonically enhanced photoemission. **a** Schematic energy diagram of the gold-vacuum interface at the emitter with present electric field *F*_pump_ = 0.5 V nm^−1^ (black line). The Fermi energy *E*_Fermi_ is ~5.1 eV below the vacuum level (dotted line). The barrier can be overcome by a multiphoton absorption (vertical dashed dotted line) or a tunneling process (horizontal dashed line). The colored lines consider the Schottky effect and a field-enhancement of 1 (blue), 2 (turquoise), 5 (green), and 10 (red). **b** SEM image of asymmetric nanojunctions with emitter (‘E’) and collector (‘C’) electrodes. Inset: numerically computed field enhancement *g* within such an asymmetric nanojunction for *E*_pump_ = 1.3 eV. **c** Polarization dependence of *I*_emission_ at *E*_pulse_ = 150 pJ and a FWHM of 14 fs of the pump pulse. Red line is a cosine fit, the gray area in the center indicates the noise level obtained without illumination

For the highest *E*_pulse_ = 320 pJ and a Gaussian pulse length *τ*_pulse_ = 14 fs, we estimate the peak electric field of the pump laser pulse to be *F*_pump_ = 0.5 V nm^−1^ in open space. For this electric field strength, Fig. 2a shows the corresponding schematic energy diagram of a gold-vacuum interface (black line). As depicted by the colored lines, the potential barrier is reduced by the Schottky effect to

1 $$W_{\mathrm {barrier}} = W_{\mathrm {gold}}-\sqrt{\left[ {e^3gF_{\mathrm {pump}}/\left( {4\pi \varepsilon _0} \right)} \right]},$$

with *W*_gold_ = 5.1 eV the work function of gold, *ε*_0_ the vacuum permittivity, *e* the electron charge, and *g* the field-enhancement factor. For instance, we compute *W*_barrier_ = 3.2 eV according to Eq. (1) for *g* = 5 at the emitter tips. Figure 2b shows a close-up SEM image of the emitter and collector structure. For *E*_pump_ = 1.3 eV, we numerically calculate a (resonant) plasmonic field-enhancement factor in the range up to *g* ~9 at the tips of the emitter, while at the collector *g* < 4 (cf. inset of Fig. 2b and Supplementary Fig. 3). This asymmetry of the nanojunctions favors the photoemission of electrons from the emitter to the collector, which explains the unipolar amplitude of *I*_emission_ in our experiments. As is typical for such plasmonic nanojunctions, their extinction strongly depends on the polarization of the exciting laser field. Indeed, we find a maximum extinction for the linear optical polarization aligned along the tips of the emitters in agreement with a dominant dipolar excitation. Consistently, the emission current *I*_emission_ follows this polarization dependence (cf. Fig. 2c).

For the highest laser field of *F*_pump_ = 0.5 V nm^−1^, the so-called Keldysh parameter *γ* can be estimated to be 2.1 for *g* = 9 and 5.5 for *g* = 4 in our experiment^27^. Both values suggest that the multiphoton absorption is the dominating mechanism in our asymmetric nanojunctions for the given laser pulses^6,7,13,14^, and strong-field photoelectron emission processes are negligible (horizontal dashed arrow in Fig. 2a)^5^. The absorption of multiple photons with a combined energy of *E*_multiphoton_ = *β* · *E*_pump_ = (1.8 – 3.9) eV is consistent with the sketched barrier heights in Fig. 2a (vertical, dashed dotted arrow). Moreover, it nicely explains the measured power coefficient *β* in Fig. 1d. We note that the amplitude of *I*_emission_ translates to only ~0.08 electrons emitted per optical pulse in average, which suggests that Coulomb repulsion and electron interference effects are negligible during the photoemission process^6,7,16^.

### Ultrafast coherent on-chip THz pulses

**Fig. 3:**
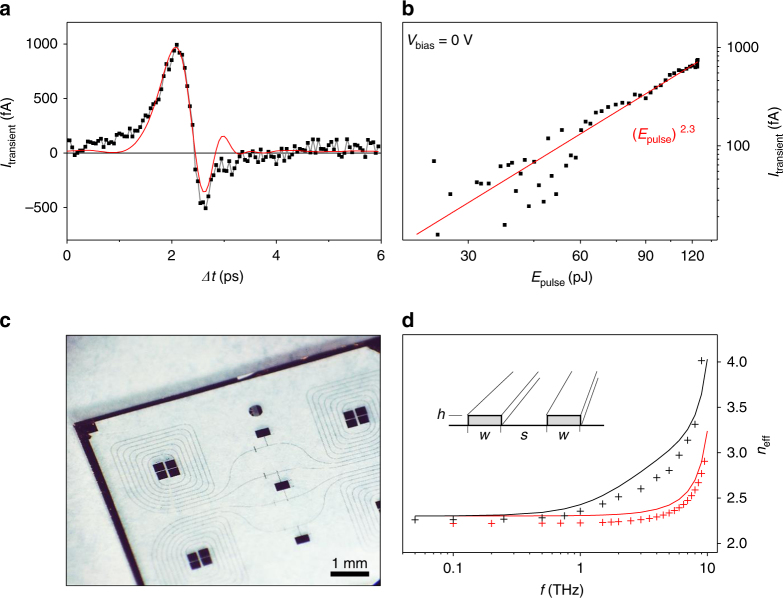
Non-linear THz pulses in macroscopic on-chip circuits. **a** Time-resolved *I*_transient_ vs *Δt* (black squares) and fit function (red line) after exciting a nanojunction integrated in the stripline circuits with a 14 fs laser pulse at *E*_pulse_ = 124 pJ. The THz signal is detected after a propagation length of 300 µm. **b** Non-linear *I*_transient_ vs *E*_pulse_ with a power law fit (*E*_pulse_)^*β*^ (red line) showing a similar power law coefficient *β* as found for *I*_emission_ (cf. Fig. 1d). **c** Microscope image of the utilized THz-circuitries on a sapphire chip. **d** Dispersion relation of the effective diffraction index *n*_eff_ of co-planar gold striplines on a sapphire substrate (inset): black (red) crosses depict numerical simulations with dimensions *h* = 300 nm, *w* = 5 µm (1 µm), and *s* = 10 µm (1 µm). The black (red) line shows an analytical solution for symmetrically spaced striplines with dimensions: *h* = 300 nm, *w* = *s* *=* 10 µm (1 µm)^31^

In the next step, we show that *I*_emission_ can drive THz pulses in stripline circuitries as sketched in Fig. 1c. After the excitation of the nanojunctions, the THz pulses run along the striplines up to several hundreds of micrometers, and are detected on-chip by the time-delayed optical probe pulse in combination with a semiconductor Auston switch^17,21–23^. The latter is made from ion-implanted amorphous silicon with a sub-picosecond (~500 fs) time resolution^21–23^. The resulting current *I*_transient_ across the Auston switch is sampled as a function of the time delay *Δt* between the pump and the probe pulse (Fig. 3a). Importantly, we find a non-linear power dependence of *I*_transient_ vs *E*_pulse_ with respect to the pump laser, when the nanojunctions are optically excited (Fig. 3b). The observed power law coefficient *β* is consistent with the one deduced for *I*_emission_ for our junctions (Fig. 1d), which demonstrates that the signal *I*_transient_ is a global read-out of *I*_emission_ along the striplines.

Generally, the signal *I*_transient_, as measured at the Auston switch, is directly proportional to the electric field component of the THz pulse as it propagates along the striplines with macroscopic dimensions (Fig. 3c)^17,18,28^. The dispersion of the striplines allows the propagation of signals up to several THz (Fig. 3d), before losses, predominantly to the Al_2_O_3_-substrate, set in^29^. Assuming an initial Gaussian THz pulse at the position of the nanojunctions, we accordingly calculate its time and space evolution along the striplines^29^. At the position of the semiconducting Auston switch, the computed pulse agrees well with the measured *I*_transient_ (red line in Fig. 3a). We note that in this calculus, the propagating THz pulse is convoluted with the (much slower) read-out time of the semiconducting Auston switch. In other words, the time resolution of our circuit is limited by the charge carrier lifetime of the utilized semiconductor Auston switch^19^. We find an apparent FWHM of the THz Gaussian to be ~500 fs (cf. Supplementary Fig. 4), which is consistent with the fastest charge carrier lifetimes in ion-implanted silicon switches reported so far^19^.

## Discussion

**Fig. 4:**
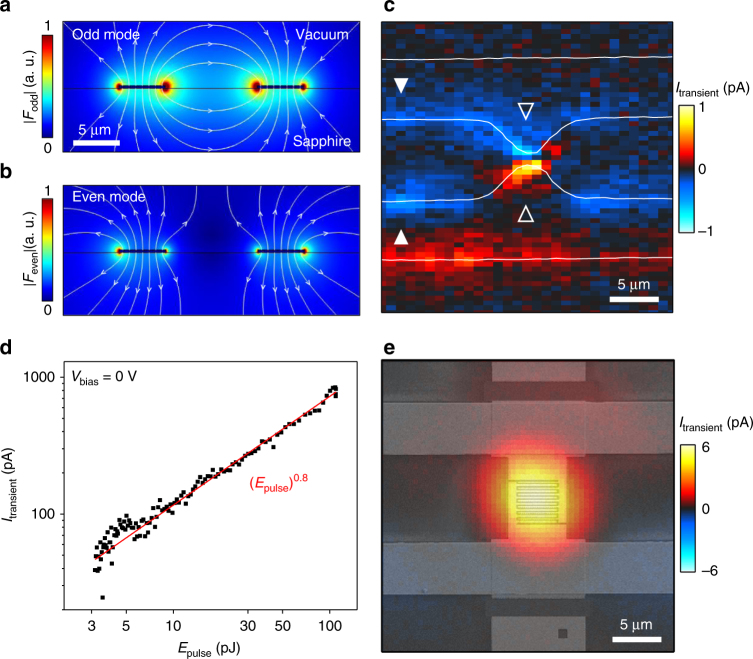
Femtosecond near-field coupling of NIR pulses to THz stripline modes. Simulated electric field distribution of the co-planar striplines (black) with **a** the odd mode and **b** the even mode. Color code describes the absolute electric field. The arrows denote the direction of the electric field vector in the image plane. **c** Spatial map of *I*_transient_ at fixed *Δt* for a sample without nanojunctions (white lines indicate the striplines). The odd mode is excited in the center with a minimum distance of 1 µm between the striplines (open triangles). The even mode is excited at edges where the striplines have a distance of 10 µm (filled triangles). **d** *I*_transient_ vs *E*_pulse_ for striplines without nanojunctions showing an almost linear dependence (red line). **e** Spatial map of *I*_transient_ for asymmetric nanojunctions integrated in the striplines with an overlaid SEM image

On first view, it is surprising that an optical pulse with 270 THz (*E*_pump_ ~1.3 eV) can be down-converted to a coherent 2 THz signal in the striplines. However, when electrons propagate ballistically across the nanojunctions, a unipolar displacement current can couple into the THz striplines by near-field interactions, despite the frequency and momentum mismatch^17^. To elucidate the near-field interactions in the stripline circuits, we depict side-cuts of the two dominant stripline modes with the odd (even) mode exhibiting an opposite (the same) polarity at each stripline (Fig. 4a, b). Intriguingly, we can visualize the two modes in our stripline circuits. To do so, we record spatial maps of the maximum *I*_transient_ for a fixed *Δt* in striplines *without nanojunctions* (Fig. 4c). Such maps reveal a signal of *I*_transient_ at all stripline edges at smaller amplitude. The polarity distribution of *I*_transient_ in Fig. 4c suggests that the odd mode is excited at the center of the striplines (open triangles). At the edges of the striplines, the even mode seems to be predominantly excited (filled triangles in Fig. 4c). At all stripline edges, *I*_transient_ follows an intensity dependence with a power law exponent *β* close to 1 or slightly below (Fig. 4d).

Generally, we would like to point out that we detect the difference of the two situations experimentally (nanojunctions exhibit a non-linear response of *I*_emission_ and *I*_transient_, and the edges of the striplines show a rather linear dependence of *I*_transient_ and the absence of a measureable *I*_emission_). Furthermore, the nanojunctions are lithographically optimized to exhibit a plasmonic field enhancement in the spectral range of the pump laser. Following earlier work^13,14^, optical rectification dominates the ultrafast THz response of metal surfaces for low laser intensities. In our understanding, such a low-intensity regime is consistent with the linear response of *I*_transient_ at the edges of the striplines (e.g., white triangles in Fig. 4c). In particular, the necessary asymmetry for an optical rectification process is naturally achieved when the laser spot is half-centered at the metallic edges (with one half on the metal and the other half on Al_2_O_3_). In our understanding, the plasmonic field enhancement at the tips of the nanojunctions shifts the local laser intensity into the regime of multiphoton-based electron emission, which allows us to measure a non-linear response of both *I*_emission_ and *I*_transient_. The essential role of photoemitted electrons in the nanojunctions is verified by the non-linear values of *β* for both *I*_emission_ and *I*_transient_, and that this value is consistent with the expected barrier profile as discussed in connection with Fig. 2. It is noteworthy that also for the striplines without nanojunctions (Fig. 4c), the center regions show a slightly increased amplitude of *I*_transient_ (open triangles) compared to all other edges of the striplines (white triangles). Most likely, this increase is caused by a field-enhancement because of the smaller distance between the two striplines at this position. An obvious question arises of whether the optical intensities there are sufficient to get into the multiphoton regime as well. For such gaps with a distance of 1.1 µm (Fig. 4c), we cannot measure any time-integrated *I*_emission_ of photoemitted electrons propagating across the gap within the given noise level (data not shown), most likely because of the insulating properties of the Al_2_O_3_ substrate. However, we performed a control experiment for such gaps with a distance of 1.1 µm to prove that also there, electrons can be photoemitted from the contacts (cf. Supplementary Fig. 5).

A second scenario considers the band alignment of the Al_2_O_3_ substrate with respect to the Fermi energy of the gold striplines. For Al_2_O_3_, the conduction band is ~4 eV above the Fermi level of Au. However, for the utilized r-plane Al_2_O_3_, surface states are reported which are aligned to the gold Fermi level within the energy bandwidth of the laser (~0.4 eV = 0.9–1.3 eV, cf. Supplementary Fig. 1)^30^. In between the Au and such localized states in the Al_2_O_3_, a thin (approximately nanometer) dielectric layer easily forms from lithographic residues, which acts as a tunneling barrier. In turn, corresponding charge tunneling processes are reasonable to be driven by the electric field of the optical pulses. Again, the necessary spatial asymmetry is realized when the laser spot is half-centered at the metallic edges. In other words, the metallic charge carrier density favors an ultrafast tunneling of electrons from the metal into the Al_2_O_3_ at the presence of the optical field. The diffusive back-flow of the transferred electrons is expected to be much slower (~ns–µs), such that this back-flow is not resolvable in *I*_transient_ within the given noise level. Within this scenario, we tentatively interpret the slight sub-linear dependence of *I*_transient_ vs intensity to stem from a saturation of the surface states occupation. An obvious question is why such tunneling processes do not show up in the signal at the nanojunctions. However, only for lithographically optimized nanojunctions, we find a non-linear power law (*E*_pulse_)^*β*^. For such nanojunctions, the plasmonic enhancement of the gap modes favors the non-linear multiphoton electron emission, which apparently dominates the saturating tunneling currents into the Al_2_O_3_ surface states. By detecting *I*_transient_, we cannot exclude an additional electron flow into the Al_2_O_3_substrate at the metal edges, but both discussed scenarios suggest a multiphoton electron emission at the nanojunctions which is verified by the detection of a unipolar, non-linear current *I*_emission_.

We note that we measured *I*_transient_ as a function of the laser polarization also for the positions as indicated by the open triangles in Fig. 4c. There, we detect a finite signal only for the shown (perpendicular) polarization. A polarization parallel to the metal edges of the striplines does not give a resolvable *I*_transient_ (data not shown). Hereby, we exclude a photo-Dember effect of charge carriers with asymmetric diffusivity for instance in the Au and Al_2_O_3_ to be a dominating process. Last but not least, we center the nanojunctions in between the striplines and orient the emitter and collector electrodes in a way to maximize the near-field interactions of the nanojunctions and the odd mode of the striplines (cf. Fig. 4a, e).

The on-chip THz pulses have an apparent FWHM of ~500 fs (cf. Fig. 3a and Supplementary Fig. 4). As mentioned in the Results section, this value is consistent with the fastest charge carrier lifetimes in ion-implanted silicon switches reported so far^19,23^. We note, however, that the unipolar, non-linear *I*_emission_ demonstrates that the photoemission of electrons in the nanojunctions is responsible for the ‘high-frequency generation’. Since the velocity of the ballistic electrons within the gap is experimentally not accessible, we take the Fermi velocity in the order of ~10^6^ ms^−1^ of the impinging electrons in the emitter as a naive lower boundary. Possible ponderomotive acceleration effects in the strong-field photoemission regime are supposed to give faster velocities^5,6^. A corresponding upper boundary of the time of flight across the 50 nm gap gives ~ 50 nm/10^6^ ms^−1^ = 50 fs, which is slightly longer than the optical pulse width of ~14 fs. The above argument makes it reasonable that the ‘high-frequency generation’ in the nanojunctions occurs on a timescale of a few tens of femtoseconds. Given the monotonic dispersion up to ~10 THz for Au striplines on Al_2_O_3_ (cf. Fig. 3d)^29,31–33^, the time resolution of *I*_transient_ is ultimately limited by the ballistic time of flight of the electrons from the emitter to the collector. For instance, shrinking the striplines’ dimensions (cf. Fig. 3d for striplines with *w* = *s* = 1 µm) and further optimization of the material parameters (e.g., glass substrate), as well as the utilization of superstrates, will be useful to push the dispersion and attenuation up to ~10 THz^31–33^.

Taking the utilized compact Er:fiber technology^25^, our results show a major step forward to combine optical and electronic circuits for telecom compatible high-frequency wafer-scale applications. We demonstrate that asymmetric plasmonic nanojunctions can drive ultrafast photoemission currents at zero bias voltage, and that such current pulses drive coherent THz transients within on-chip high-frequency circuits. Moreover, the transients can be measured up to a millimeter propagation length. We present detailed insights into the femtosecond near-field coupling mechanisms between optical and THz pulses (cf. Fig. 4) and we provide a theoretical understanding of the dispersion that presents the main limitation for the macroscopic on-chip propagation of the THz signals within stripline circuits. Decreasing the nanojunctions’ gap and the corresponding time of flight of photoemitted electrons across the gap can further enhance the fundamental temporal response of the nanojunctions. We note that by introducing the asymmetric nanojunctions in this work (Fig. 2b), we achieve the required spatio-temporal symmetry breaking which is necessary to induce unipolar photoemission currents in nanoscale circuits. The advantage of this approach is that we can detect ultrafast photoemission currents even for laser pulse durations of up to 20 fs (cf. Fig. 1e) without the need for few-cycle (asymmetric) femtosecond laser systems. This presents an encouraging step towards non-linear, wafer-scale femtosecond electronics.

## Methods

### Fabrication of the asymmetric nanojunctions

As substrate we use sapphire with a thickness of 340 µm, covered with 300 nm silicon. The silicon is implanted with O_2_ to yield an excess carrier lifetime of ~500 fs. In a first lithographical step, we etch the silicon using HF/HNO_3_ to form the Auston switches. In two subsequent optical lithography steps, we first evaporate a Ti/Au film of 2/35 nm for the nanojunctions and then the Ti/Au striplines with 10/300 nm. The asymmetric nanojunctions are fabricated using FIB milling of the Ti/Au film which is located in a distance of ~350 µm to the Auston switch. The striplines have a total length of ~48 mm and are separated by 10 µm.

### Time-integrated photocurrent spectroscopy

We measure the time-integrated *I*_emission_ by raster-scanning the pump laser across the area of the nanojunctions at a chopper frequency of 6 kHz using a lock-in amplification scheme. We use an Er-fiber-based pulsed laser (repetition rate 80 MHz) as pump. The pump pulses pass through a non-linear fiber and two SF10-prism pairs to tune the broadband spectrum (0.9–1.3) eV as well as the pulse length (>14 fs) with a maximum average laser power of 55 mW. The pump pulses are focused on the nanojunctions by a CaF_2_ lens (*f* = 40 mm) to a spot size of ~7.5 µm (FWHM). We achieve identical experimental results also with a refractive objective. All measurements are done in vacuum (10^−6^ mbar) at *T* = 77 K.

### On-chip time-domain terahertz spectroscopy

For the time-resolved measurements, we utilize the same laser for the pump as for the time-integrated photocurrent measurements (see above Methods section). After excitation by the pump pulse, the ultrafast field emission current across the asymmetric nanojunctions couples into the striplines. Consequently, an electromagnetic transient, proportional to the initial current, propagates along the striplines. After a time delay *Δt*, the probe pulse triggers the Auston switch. The probe pulses have a pulse duration of 100 fs, energy of 1.59 eV, laser power of 80 mW, and are focused on the Auston switch by a 10× objective. The spot size of the probe laser is chosen in a way to maximize the read-out signal and yields 9 µm. The presence of the electromagnetic transient at the switch drives the current *I*_transient_(*Δt*) to the read-out contact. We use frequency modulation of the pump laser in combination with a lock-in amplifier for the read-out. All measurements are done in vacuum (10^−6^ mbar) in a cryostat. The measurements are done at 77 K to minimize ohmic losses and the skin depth of the THz modes in the striplines.

### Simulation of plasmonic enhancement of the nanojunctions

To simulate the field enhancement of the nanojunctions, we apply finite element simulations using COMSOL Multiphysics®. The model for the simulation in Fig. 2b consists of a gold nanojunction with height 37 nm on a sapphire substrate. We calculate the scattering cross-section of an incident light beam as well as the generated electric field distribution in the nanojunctions to deduce the field enhancement at the emitter tips and the collector. In particular, we follow the work by ref. ^34^ neglecting both specific facets of the gold electrodes in the nanojunctions and possible local changes of the work function^16^. In a first step, the background electrical (optical) field is analytically calculated solving the Fresnel equations for the vacuum-substrate interfaces without considering the metal structures on top. In a second step, the metal structures are included for calculating the scattering amplitude induced by the plasmonic nanojunctions. Hereby, we can compute the field-enhancement distribution of the nanojunctions and the stripline edges. Further details, e.g., for diffraction index of the substrate and the gold, are given in supplementary Fig. 3.

### Simulation of the THz dispersion

To calculate the THz dispersion, we use COMSOL 3D and 2D frequency domain simulations of gold striplines with height *h* = 300 nm and different values for the width *w* and separation *s* (cf. Fig. 3d). We simulate the propagation of plane waves with different frequencies along the striplines to get the dispersion relation of the effective refractive index *n*_eff_. Gold is considered to be a ‘perfect conductor’ within the COMSOL database, and the dielectric function of Al_2_O_3_ is taken from refs. ^32,33^

### Data availability

All relevant data that support our experimental findings are available from the corresponding author upon reasonable request.

## Electronic supplementary material

Supplementary Information
