# Supplementary material for: Towards femtosecond on-chip electronics based on plasmonic hot electron nano-emitters
Source: Nat Commun. 2018 Jun 25;9:2471. doi: 10.1038/s41467-018-04666-y (PMC6018641; doi:10.1038/s41467-018-04666-y)
Supplement: Supplementary file 1 — Supplementary Information [file 41467_2018_4666_MOESM1_ESM.pdf]

Towards femtosecond on-chip electronics based on plasmonic hot electron nano-emitters

Karnetzky et al.

Supplementary information

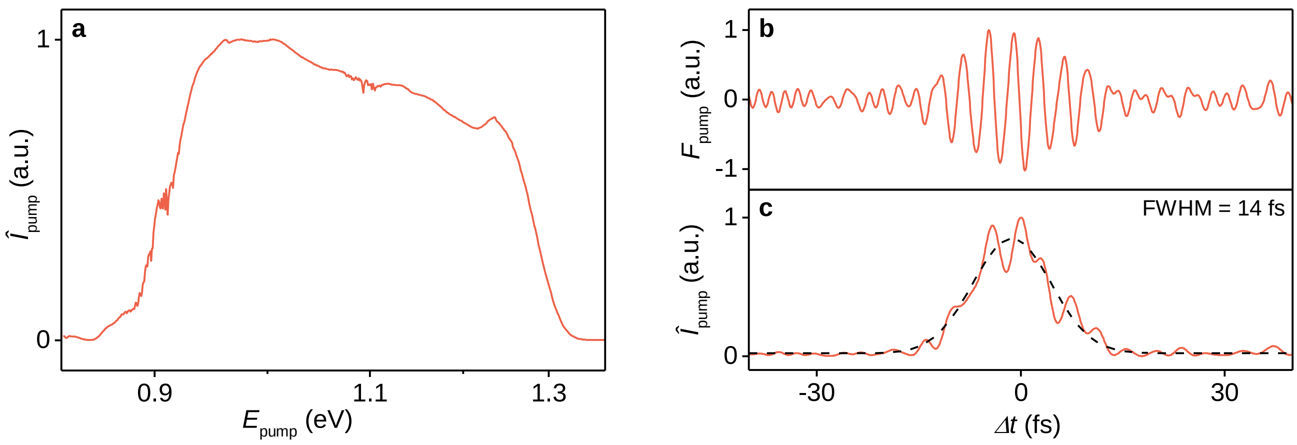

Supplementary Figure 1 | Frequency resolved optical gating of the utilized laser pulse. a, Pump laser intensity $\hat{I}_{pump}$ vs. E_{pump} of the utilized pump laser. **b,** Electric field F_{pump} and **c,** pump laser intensity $\hat{I}_{pump}$ of the shortest pulses vs time delay Δt retrieved from the phase-resolved SHG-FROG characterization.

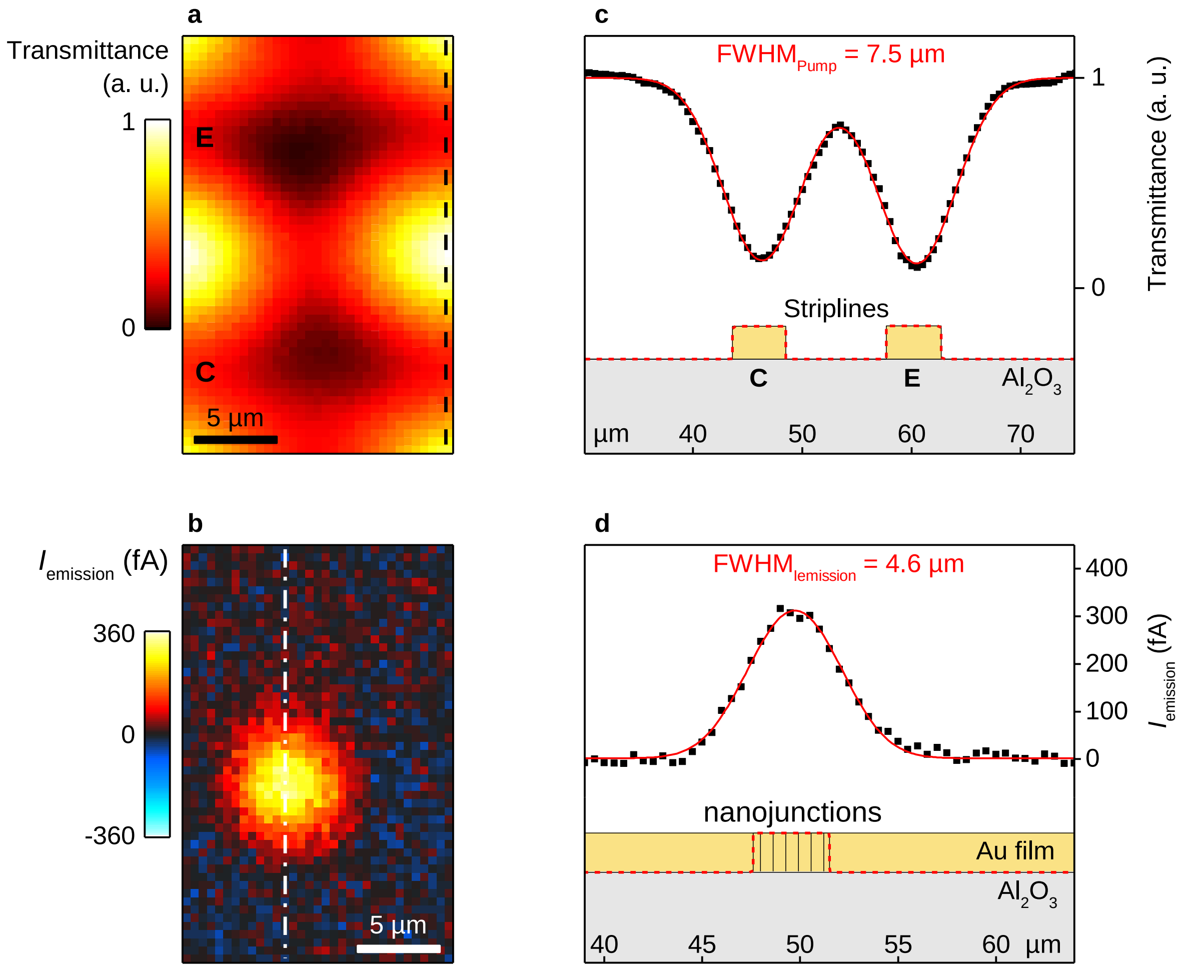

Supplementary Figure 2 | Spatial photocurrent mapping. Spatial profiles of **a**, the transmittance through the Al_2O_3-substrate and **b,** the simultaneously measured $I_{emission}$, while the pump laser is scanned across the sample. Clearly, one can identify the emitter 'E' and the collector 'C' of the striplines with the centered nanojunctions (compare Fig. 1b in the main manuscript). **c,** Transmittance profile along the dashed line in (a). The fit function to the transmittance (red line) is the convolution of a multistep function representing the stripline geometry (dashed red line in lower part) and a Gaussian with $FWHM_{Pump}$ = 7.5 µm according to the optical resolution of the pump laser. **d,** Profile of $I_{emission}$ along the dashed-dotted line in (b). The fit function (red line) is the convolution of a double-step function representing the area of the nanojunctions (dashed red line in lower part) and a Gaussian. The corresponding $FWHM_{Iemission}$ = 4.6 µm is significantly narrower than the above defined optical resolution $FWHM_{Pump}$ of the pump laser.

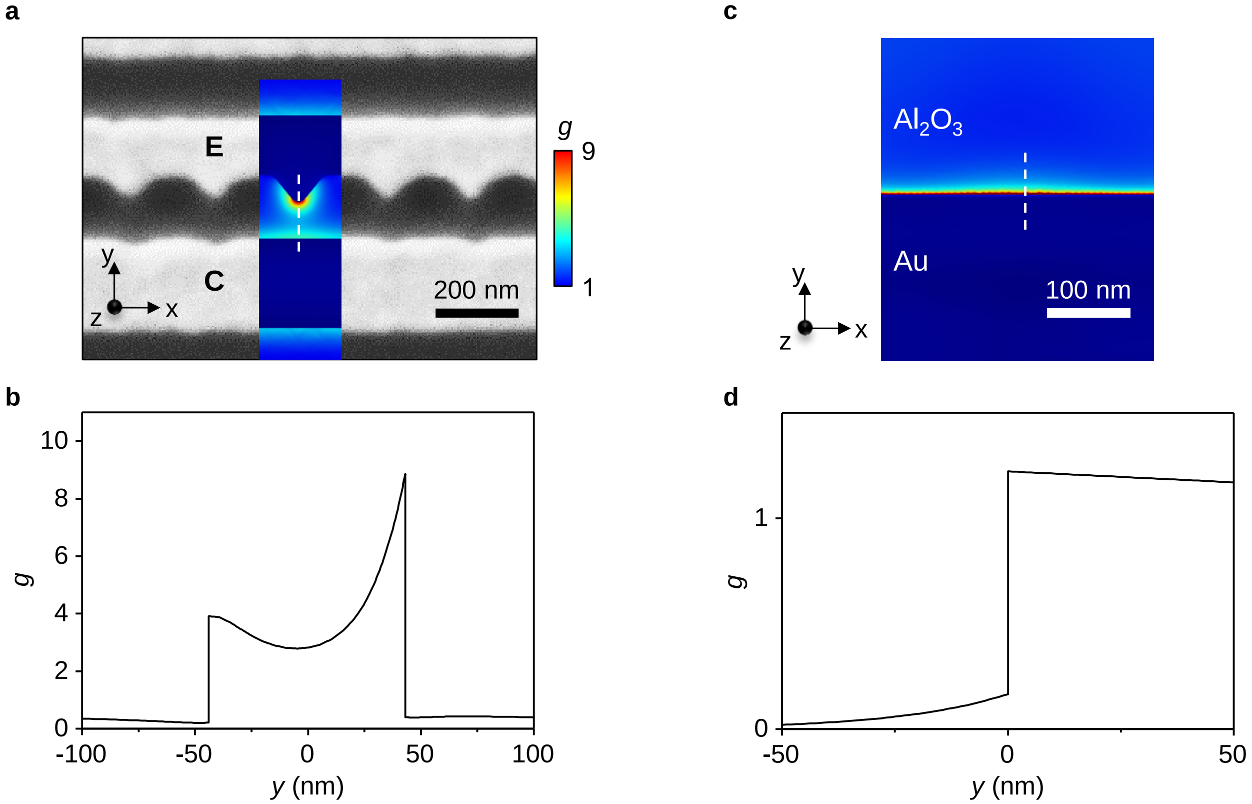

Supplementary Figure 3 | **a,** Same figure as Figure 2b of the main manuscript. **b,** Field enhancement *g* along the white dashed line in (a) for E_{pump} = 1.3 eV. The maximum field enhancement *g* is between 4 and 9 at the opposing tips of the asymmetric nanojunctions. **c,** Simulated field enhancement for a stripline edge made out of Au on Al_2O_3 without nanojunctions. **d**, Field enhancement along the dashed line in (c) for E_{pump} = 1.3 eV. The dielectric functions for Al_2O_3 and gold are taken from Supplementary References [1] and [2].

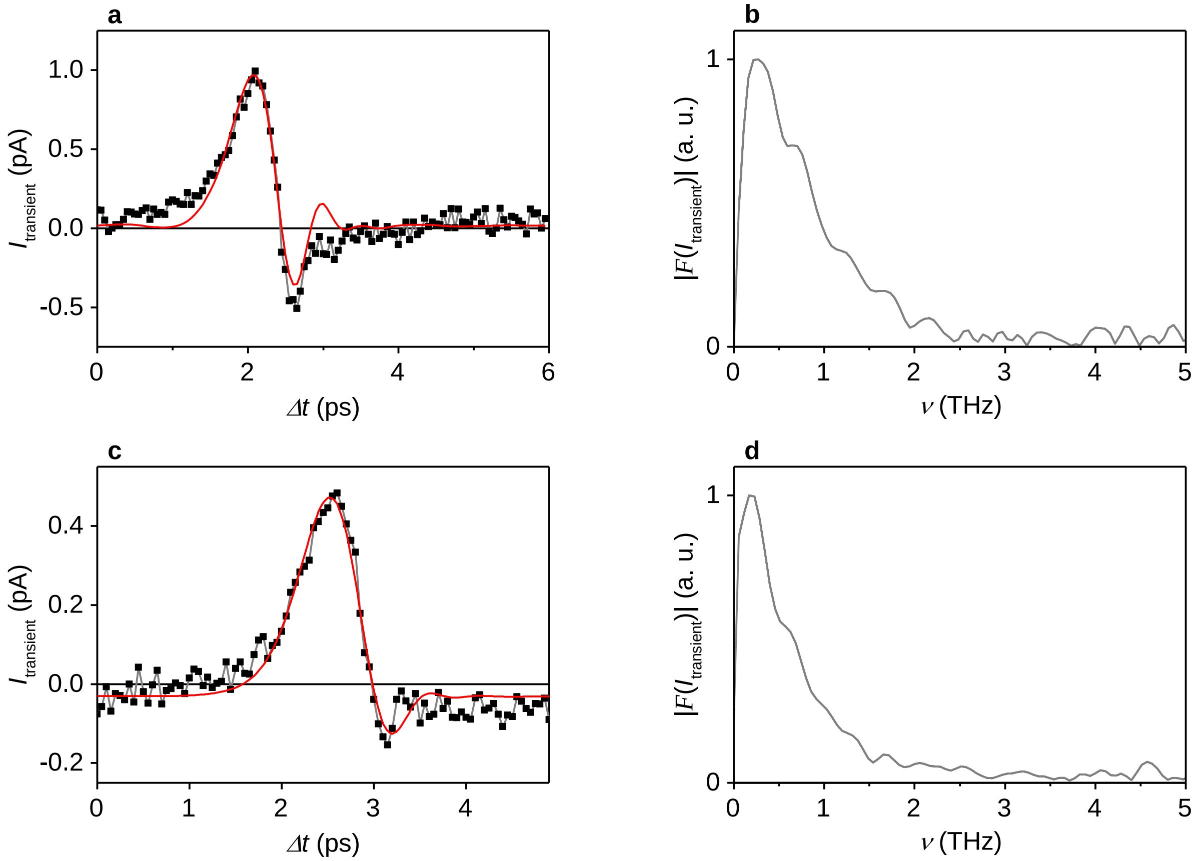

Supplementary Figure 4 | **a,** Same figure as Figure 3a of the main manuscript: Time-resolved $I_{transient}$ vs time delay Δt (black) and fit function (red) after exciting a nanojunction integrated in the stripline circuits with a 14 fs laser pulse at E_{pulse} = 124 pJ. The THz-signal is detected after a propagation length of 300 µm. Red line is a fit as described in the main manuscript. **b,** Fourier transform of the data of (a) showing Fourier-coefficients up to ~2 THz. **c,** $I_{transient}$ vs Δt for an excitation position of the pump laser at the edges of the striplines (open triangles in Fig. 4b of the main manuscript). The transient has an initial FWHM of 500 fs, which is consistent with the time-scale given by the non-radiative lifetime of the semiconductor Auston switch. **d,** Fourier transform of the data in (c) showing Fourier-coefficients up to ~2 THz.

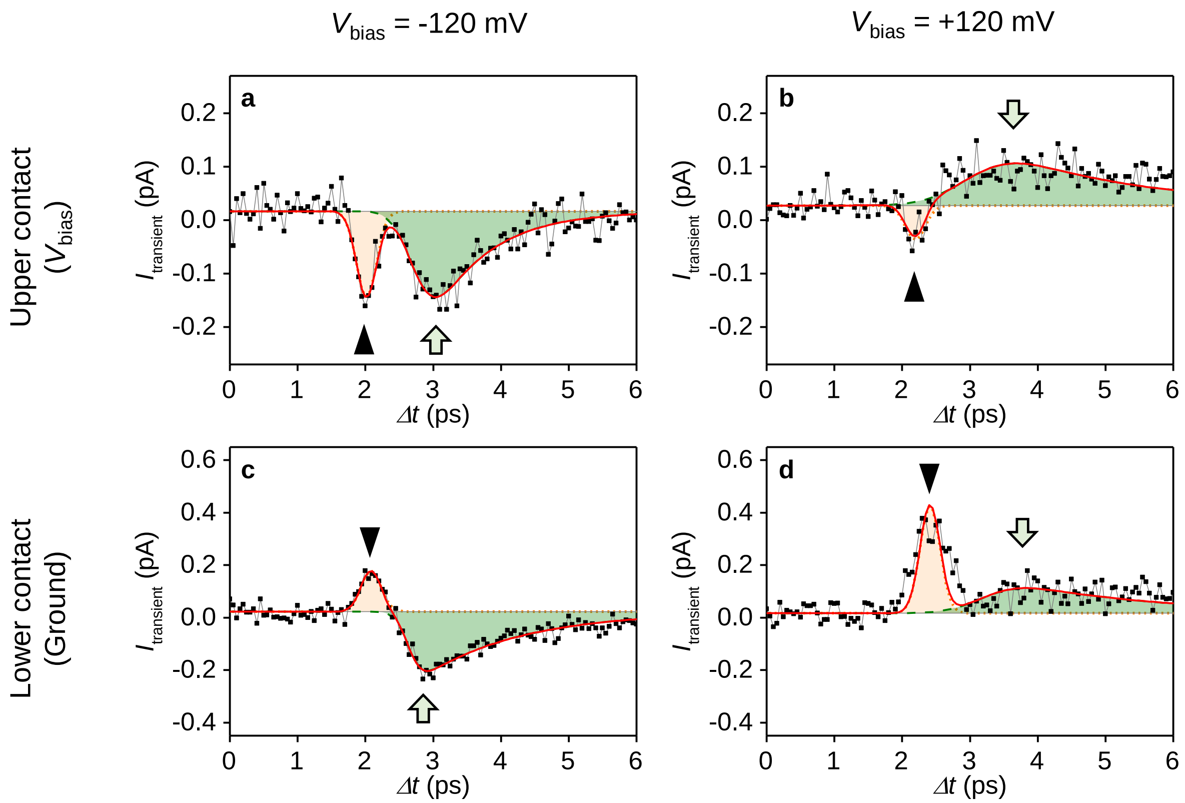

Supplementary Figure 5 | Time-resolved photocurrents in carbon nanotubes spanning striplines with a distance of 1 µm. For the plasmonic nanojunctions, the time-integrated, unipolar $I_{emission}$ is a clear evidence of the photoemitted electrons (Fig. 1b). For the gaps with a distance of 1.1 µm (Fig. 4b), we do not measure any time-integrated $I_{emission}$ within the given noise level (data not shown), most likely because of the insulating properties of the Al_2O_3-substrate. However, we performed a control experiment for such gaps with a distance of 1.1 µm. We integrated individual, semiconducting carbon nanotubes (CNTs) into such gaps (cf. Supplementary Reference [3]). The CNTs solve two purposes in this control experiment; first, that indeed, we can detect a (photogenerated) charge current across the gap (also at zero bias), and second, that the applied bias drops at the nanometer footage of the CNTs for low voltages. In other words, the electrostatic fields, which are relevant for the photoemission processes, are enhanced. The CNTs are resonantly excited by the same laser as in the main manuscript. Panels **a**, and **b**, show corresponding time-resolved photocurrent measurements when the laser is focused on one of the contacts (named upper contact) with an applied bias V_{bias} = -120 mV and +120 mV. Again, the striplines and the corresponding circuit are quasi identical to the ones as for Fig. 4b. We clearly can distinguish two peaks. The second peak (arrows and green shading) can be identified to stem from non-equilibrium, ballistic currents in the CNTs (as discussed in detail in Supplementary Reference [3]). The first peak (black triangles and red shading) is consistent with electrons stemming from a photoemission process at the upper contact. In particular, the amplitude of the first peak decreases with increasing V_{bias}, but it is finite at zero bias (Supplementary Reference [3]). Moreover, at the grounded lower contact (Panels **c** and **d**), the first peak changes sign and its amplitude increases with increasing V_{bias}.

Both observations are consistent with electrons, which are photoemitted from the metallic contacts into the CNTs and propagating to the other contact. An optical rectification process would not depend on the bias voltage V_{bias}. Furthermore, the sign change is consistent with the sign change of $I_{transient}$ as measured without CNTs at the two inner contacts in Fig. 4b (open triangles). We note, however, that for the experiment with the CNTs hot carrier effects are involved (cf. Supplementary References [3],[4]) and at low laser powers, also an optical rectification is most likely involved.[5] Experimental parameters are $\hat{I}_{laser}$ = 23 kW cm^{-2} , and temperature T = 77 K.

Supplementary References:

1. Malitson, I. H. & Dodge, M. J. efractive index and birefringence of synthetic sapphire. *J. Opt. Soc. Am.* **62,** 1405 (1972).

2. Johnson, P. B. & Christy, R. W. Optical Constants of the Noble Metals. *Phys. Rev. B* **6,** 4370–4379 (1972).

3. Karnetzky, C., Sponfeldner, L., Engl, M. & Holleitner, A. W. Ballistic and resonant negative photocurrents in semiconducting carbon nanotubes. *Phys. Rev. B* **95,** 161405 (2017).

4. Herink, G., Wimmer, L. & Ropers, C. Field emission at terahertz frequencies: AC-tunneling and ultrafast carrier dynamics. *New J. Phys.* **16,** 123005 (2014).

5. Ramanandan, G. K. P., Ramakrishnan, G., Kumar, N., Adam, A. J. L. & Planken, P. C. M. Emission of terahertz pulses from nanostructured metal surfaces. *J. Phys. D: Appl Phys.* **47**, 374003 (2014).
